# Cross-cultural validation of the stroke riskometer using generalizability theory

**DOI:** 10.1038/s41598-021-98591-8

**Published:** 2021-09-24

**Authors:** Oleg Medvedev, Quoc Cuong Truong, Alexander Merkin, Robert Borotkanics, Rita Krishnamurthi, Valery Feigin

**Affiliations:** 1 School of Psychology, Faculty of Arts and Social Sciences, University of Waikato, Private Bag 3105, Hamilton, 3240 New Zealand; 2 Auckland University of Technology, School of Clinical Sciences, Auckland, New Zealand

**Keywords:** Neuroscience, Diseases, Health care, Neurology, Risk factors

## Abstract

The Stroke Riskometer mobile application is a novel, validated way to provide personalized stroke risk assessment for individuals and motivate them to reduce their risks. Although this app is being used worldwide, its reliability across different countries has not yet been rigorously investigated using appropriate methodology. The Generalizability Theory (G-Theory) is an advanced statistical method suitable for examining reliability and generalizability of assessment scores across different samples, cultural and other contexts and for evaluating sources of measurement errors. G-Theory was applied to the Stroke Riskometer data sampled from 1300 participants in 13 countries using two-facet nested observational design (person by item nested in the country). The Stroke Riskometer demonstrated strong reliability in measuring stroke risks across the countries with coefficients *G* relative and absolute of 0.84, 95%CI [0.79; 0.89] and 0.82, 95%CI [0.76; 0.88] respectively. D-study analyses revealed that the Stroke Riskometer has optimal reliability in its current form in measuring stroke risk for each country and no modifications are required. These results suggest that the Stroke Riskometer’s scores are generalizable across sample population and countries permitting cross-cultural comparisons. Further studies investigating reliability of the Stroke Riskometer over time in longitudinal study design are warranted.

## Introduction

Stroke is the second leading cause of death and the third leading cause of disability worldwide^1^. The number of incident strokes has been increasing dramatically from approximately 7.2 million people in 1990 to 12.2 million in 2019 (69% increase), while the number of stroke survivors over that time period has increased from 54.7 million to 101.5 million (86%)^1^. Stroke is no longer a disease of older people like it was 30–40 years ago, with a significant increase in incidence rates in people 20–54 years old. Now more than 60% of people affected by stroke are younger than 75 years^2^. These data indicate that the burden of stroke is more likely to surge in the future even though stroke is highly preventable^2^ and that currently used primary stroke prevention strategies are not sufficiently effective^3^. If more affordable and effective prevention strategies were implemented, the stroke burden could be substantially decreased.

**Figure 1:**
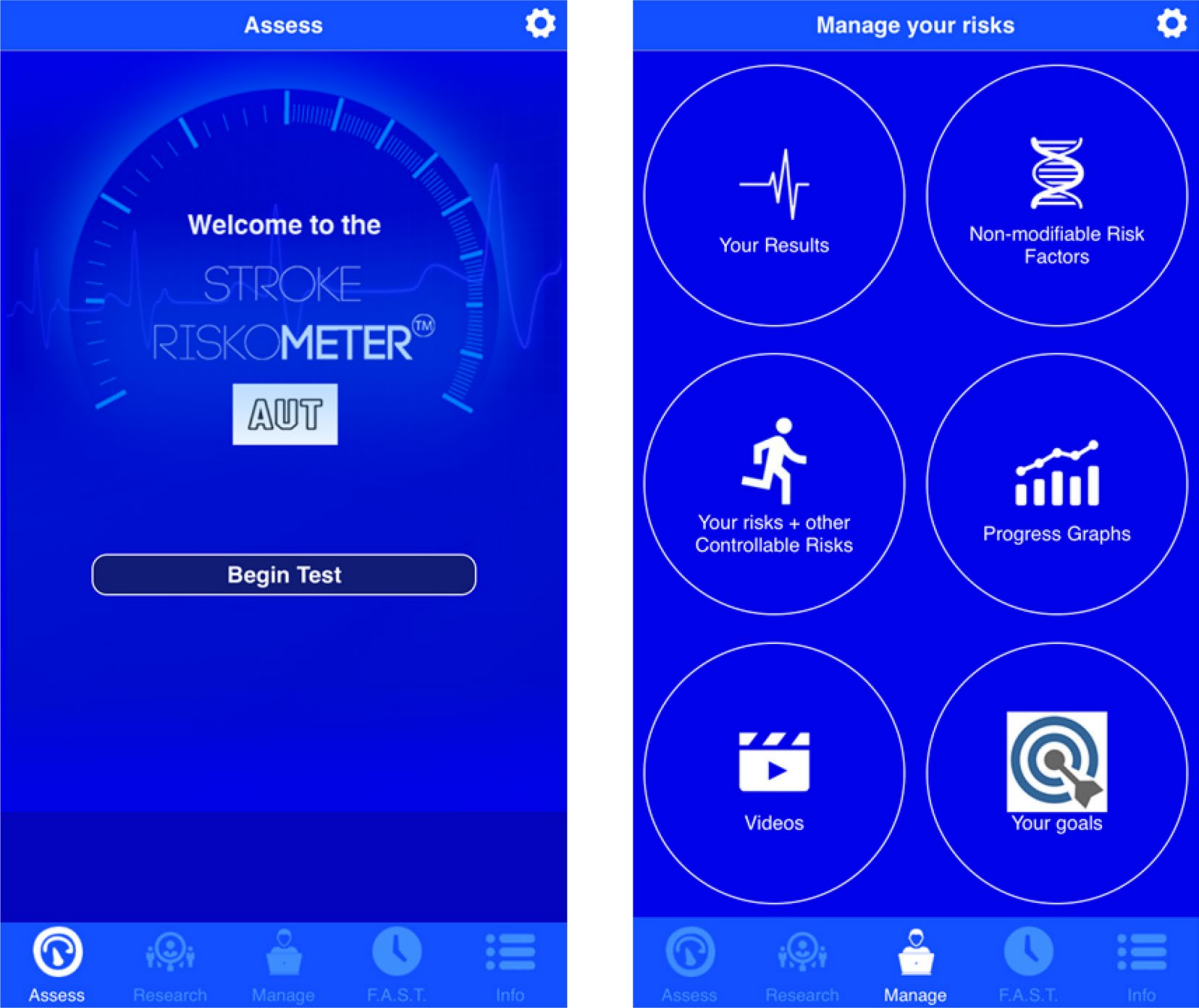
Stroke Riskometer mobile application front page.

Currently, personal modern technologies (e.g., Smartphones) are used worldwide with increasing numbers of users, allowing increased productivity and convenience. One of the important advantages of far reaching mobile health applications (apps) is that they can offer personalized assessments that may provide novel opportunities to enhance individuals’ health and reduce the burden of many diseases including stroke^4,5^. Mobile apps can be both accessible and cost‐effective (free to use). There is evidence that using the relevant Smartphone applications elicit behavior change in preventing stroke^4,5^. The National Institute for Stroke and Applied Neurosciences (NISAN) at Auckland University of Technology (AUT University) has developed a free to use mobile app called the Stroke Riskometer^6^. Figure 1 shows the front page of the app, which utilizes the international guidelines on stroke prevention to increase awareness about the disease and its risk factors on an individual level^7^. The algorithm which is used in the Stroke Riskometer was adapted from the Framingham Stroke Risk Score (FSRS) prediction algorithm^8^ by adding additional important (primarily lifestyle) risk factors and properly validated^9^.

The Stroke Riskometer comprises 21 assessment items including three demographic questions (i.e. age, sex, and ethnicity/race), two physical questions (height and weight) and 16 questions about other major risk factors of stroke including blood pressure^6,10^. The application computes absolute stroke risk estimates in 5- and 10-year perspective for users aged 20–93. Importantly, this application also provides the relative risk calculation for stroke, which allows users to compare their stroke risk against a person of the same demographic characteristics such as age, sex and ethnicity without contributing risk factors. Relative risk estimates of the app were shown to be acceptable and motivational to the user to know their risk factors and how to control them^3,11^, thus allowing stroke prevention interventions regardless of the level of risk as recommended by the World Stroke Organization and the World Heard Federation^12^. The Stroke Riskometer covers all stroke risk levels and alerts users to reduce their risk of stroke^9^.

The Stroke Riskometer was validated by Parmar et al.^9^ using Classical Test Theory (CTT) methods. In that study, the authors explored the predictive validity of this application to stroke risk by comparing the use of this application to the other two common methods (i.e. FSRS and QStroke). The results of the study indicated that the Stroke Riskometer is as accurate as FSRS and QStroke^13^ in predicting stroke^9^. A recent pilot randomized controlled trial (RCT) conducted by Krishnamurthi et al.^11^ to examine feasibility and preliminary efficacy of the Stroke Riskometer (n = 26) app for primary stroke prevention compared to usual care (n = 24) demonstrated a high acceptability and clinically significant efficacy of the intervention to improve cardiovascular health 6 months post-randomization.

Even though the Stroke Riskometer is a novel stroke prevention tool with growing evidence supporting its reliability and validity in estimating stroke risks, this Smartphone application should be continually developed and validated to improve the accuracy of its scoring systems^9^. In addition, this application was developed in New Zealand, but its users are located worldwide. Therefore, research should assess the psychometric properties of this application across countries to establish robustness of the instrument by utilizing an appropriate methodology such as Generalizability theory (G-Theory)^14,15^.

G-Theory represents an extension of the widely used CTT methodology in examining the reliability, evaluating sources of measurement error and establishing generalizability of assessment scores^15–19^. While CTT considers that error variance of any measurement is a single source, G-Theory estimates all potential sources of error variance that may influence the accuracy of the measurement^14,15,19^. CTT methods examine reliability of only one aspect of a measurement at a time (e.g. internal consistency Cronbach’s alpha or test–retest), which reflects a reliable measure with a coefficient of above 0.70^20^. Thus, CTT methods do not simultaneously consider specific measurement errors due to different sources of variability. In contrast, G-Theory closely examines all potential sources of error variance that may influence reliability simultaneously^14,16,17^. These variance sources may include effects of person, country, instrument items used in each country, and interaction effects of person and country and person and item within a specific country^15^. Therefore, G-Theory provides a more robust approach to evaluate precision of psychometric measurements across different situations and contexts (e.g. across cultures/countries). To date, G-Theory has been applied to establish reliability and generalizability of assessment scores across many different disciplines including medicine^21^, rehabilitation^22^, psychology^17^, psychiatry^23^ and education^18^.

The Stroke Riskometer app used in non-English speaking countries was translated into the languages of those countries, but the algorithm remained the same as the English version of the app. Therefore, the perception of the translated questions in the app might be different in people of different countries due to possible translation inaccuracy and/or different cultural norms. Thus, cross-country validation of the Stroke Riskometer is important and adds to the validity of the mobile tool. Currently, there is limited evidence to support reliability, validity and generalizability of the Stroke Riskometer app scores in measuring the risk of stroke across different countries. The aim of the present study was to use G-Theory analyses to assess reliability of the Stroke Riskometer and generalizability of its assessment scores across countries, and evaluate potential sources of measurement error. The application of G-Theory in this study involved a Generalizability study (G-study) and a Decision study (D-study). The purpose of the G-study was to evaluate the overall generalizability and sources of error variance of the Stroke Riskometer. The D-study was subsequently conducted to examine psychometric properties of individual items of the scale and evaluate reliability of the scale for each country, as well as the impact of one or more countries on the overall reliability of the Stroke Riskometer^15,29^.

## Results

### G-Study

Table 1 presents G-study results including the overall reliability and generalizability of scores across people, items nested in countries and countries. Both relative and absolute G coefficients were 0.84 and 0.82 (*Gr* and *Ga*, respectively), indicating strong reliability. The results showed that there were only two main sources of error variance. After accounting for the true variance of person, the largest source of error was the interaction between person and item nested in country Px(I:C), which accounted for 84.3% of the total error variance following by item nested in country (I:C) that explained the remaining 15.7% of measurement error in the current design.

**Table 1:** G-study estimates for the Stroke Riskometer including standard errors (SE), grand mean (GM), standard error of the grand mean, Coefficient G relative (Gr), Coefficient G absolute (Ga), and variance components for the design of P × (I:C) (n = 1300).

Source of variance	Differentiation variance	Source of variance	Relative error variance	% Relative	Absolute error variance	% Absolute
P	0.004		…		…	
	…	(I:C)	…		0.000	15.7
	…	C	…		(0.000)	0.0
	…	P × (I:C)	0.001	100.0	0.001	84.3
	…	P × C	(0.000)	0.0	(0.000)	0.0
Sum of variances	0.004		0.001	100%	0.001	100%
Standard deviation	0.064		Relative SE: 0.028		Absolute SE: 0.030	
Coefficient G relative	0.84	95% CI [0.79; 0.89]	Grand mean for levels used: 0.227			
Coefficient G absolute	0.82	95% CI [0.76; 0.88]	Standard error of the grand mean: 0.014			

### D-Study

**Figure 2:**
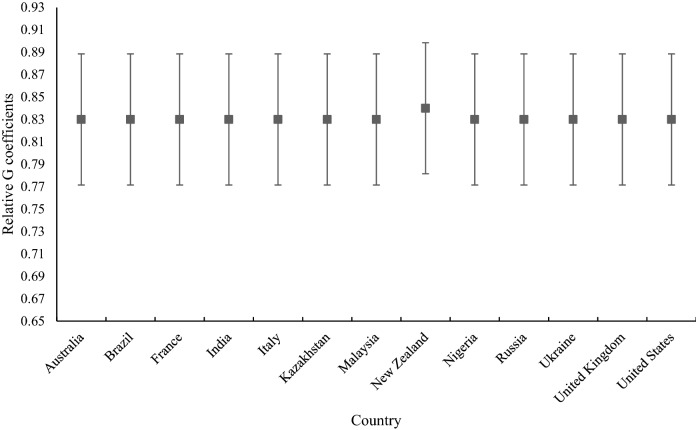
Graphical representation of reliability by relative G coefficient for each country including 95% confidence intervals.

**Figure 3:**
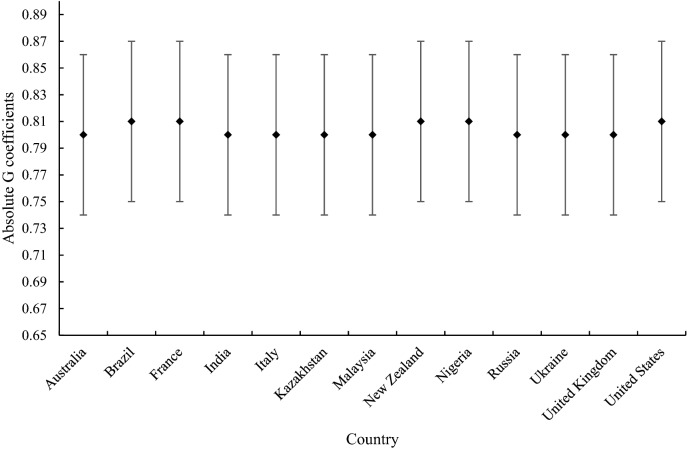
Graphical representation of reliability by absolute G coefficient for each country including 95% confidence intervals.

Figures 2 and 3 display relative and absolute *G* coefficients of the Stroke Riskometer respectively, computed for each country including 95% confidence intervals. As can be seen, reliability was consistently high across the countries. *Gr* ranged from 0.83 to 0.84 and *Ga* ranged between 0.80 and 0.81 for all thirteen countries demonstrating strong cross-cultural reliability and generalizability of the Stroke Riskometer scores. As expected, all *Ga* were slightly lower with larger confidence intervals compared to *Gr* for all countries but the ranges of 95% confidence intervals of both *Ga* and *Gr* for all countries were acceptable, further supporting reliability and generalizability of assessment scores across persons, items and countries. For instance, all lower bounds were above 0.76 for *Gr* and above 0.73 for *Ga*.

We also examined the impact on the overall reliability of the scale after excluding a specific country and some countries randomly. There were only negligible differences in overall reliability estimates when conducting G-analyses after removing one country at a time, but all *G* coefficients were 0.80 or above, which is considered a good reliability. However, reliability coefficients decreased when we randomly removed more than one country at a time. When about half of the countries were removed (6 out of 13), *Gr* dropped to the level of 0.72 which is still acceptable for trait measures (Table 3). *G* coefficients computed for individual countries were acceptable, indicating that cross-cultural validation requires at least 10 countries to ensure robustness of the results. In addition, we then conducted analyses to examine the impact of ethnicities on the overall reliability of the scale. We analysed subsamples representing Caucasians including France, Italy, Russia, United Kingdom, Australia, Ukraine, New Zealand and the United States and non-Caucasians including Brazil, Kazakhstan, Malaysia, Nigeria and India. Generalizability coefficients from these subsamples range from 0.79 to 0.82 suggesting acceptable generalizability across these two general ethnic groups. Such minor differences in coefficients found between Caucasians and non-Caucasians are likely to be due to reduced number of facets (e.g. country) and different sample sizes because G-coefficients are sensitive to the number of facets and sample size. We also investigated the influence of sex, age, weight and height on the Stroke Riskometer assessment scores with the full sample. The results indicated no significant effects of these factors on assessment scores across countries and consequently no effect on G-coefficients reflecting the reliability of the instrument. These outcomes together with other D-study results indicate that the Stroke Riskometer is reliable in estimating stroke risk with assessment scores are generalizable across people, items and countries in the current sample.

Table 2 illustrates an additional series of generalizability analyses by excluding one item at a time for the Stroke Riskometer—we examined if this might result in improved reliability of the scale in measuring stroke risks. D-study results of these analyses include variance components attributed to person, item nested in country, and their interaction together with reliability estimates *Gr* and *Ga.* The first analysis was conducted by subtracting the first item (item 1), with subsequent analyses subtracting the next item from all other items. The results showed that the main source of error variance across all analyses was the interaction between person and item nested in country which accounted for more than 83.8% of the total error variance not including the true variance of person. In addition, we also conducted further analyses to examine the impact on the overall reliability of the scale after randomly excluding some items at a time and found that the more items excluded, the lower the reliability coefficients (Table 3). There was no noticeable improvement of G-coefficients observed across analyses of the Stroke Riskometer indicating that removing any item did not improve its reliability, or even make it slightly worse. These results suggest that the Stroke Riskometer is the most reliable with its current measurement design including all 16 items tested.

**Table 2:** D-study reliability estimates and variance components for the Person (P) × Item (I): Country (C) design including interactions for the Stroke Riskometer with removing one item at a time.

Removing item	P	(I:C)		P × (I:C)		*G*_*r*_	*G*_*a*_
	σ^2^	σ^2^	%	σ^2^	%		
1	0.004	0.000	15.5	0.001	84.5	0.84	0.81
2	0.004	0.000	15.7	0.001	84.3	0.83	0.80
3	0.004	0.000	15.7	0.001	84.3	0.83	0.80
4	0.004	0.000	16.1	0.001	83.9	0.83	0.81
5	0.004	0.000	16.0	0.001	84.0	0.83	0.80
6	0.004	0.000	15.8	0.001	84.2	0.84	0.81
7	0.004	0.000	15.7	0.001	84.3	0.83	0.80
8	0.004	0.000	15.8	0.001	84.2	0.83	0.81
9	0.004	0.000	15.5	0.001	84.5	0.83	0.81
10	0.004	0.000	15.9	0.001	84.1	0.83	0.80
11	0.004	0.000	15.6	0.001	84.4	0.83	0.80
12	0.004	0.000	15.8	0.001	84.2	0.83	0.81
13	0.004	0.000	15.8	0.001	84.2	0.83	0.80
14	0.004	0.000	15.8	0.001	84.2	0.83	0.81
15	0.004	0.000	16.2	0.001	83.8	0.84	0.81
16	0.004	0.000	15.2	0.001	84.8	0.84	0.81

**Table 3:** D-study reliability estimates and variance components for the Person (P) × Item (I) : Country (C) design including interactions for the Stroke Riskometer with removing some countries or some items at a time.

Randomly removed	P	(I:C)		P × (I:C)		*G*_*r*_	*G*_*a*_
	σ^2^	σ^2^	%	σ^2^	%		
2 countries	0.003	0.000	18.7	0.001	81.3	0.80	0.76
3 countries	0.003	0.000	18.9	0.001	81.1	0.80	0.76
6 countries	0.003	0.000	19.2	0.001	80.8	0.72	0.67
4 items	0.004	0.000	20.1	0.001	79.9	0.81	0.79
8 items	0.003	0.000	18.3	0.001	81.7	0.78	0.74
12 items	0.004	0.001	17.9	0.003	82.1	0.59	0.55

## Discussion

The aim of this study was to examine the reliability of the Stroke Riskometer across countries using G-Theory. The results showed that the Stroke Riskometer is reliable and consistent in estimating stroke risk across 13 countries with G-coefficients of 0.80 and higher, meaning that the scores assessed by the Stroke Riskometer are generalizable across persons, items and countries. Our results also indicated that the scores were mainly affected by measurement error due to interaction between person and item nested in country (84.3%), which represented the highest percentage of the error variance in this study. This error may be attributed to translation of items into different languages and cross-cultural differences in interpreting specific items by completing assessments without health professional advice. The remaining 15.7% of measurement error was explained by items used in different countries. However, the impact of these errors on the overall strong reliability of the Stroke Riskometer was negligible with all G-coefficients of 0.80 or above^15,24^.

The Stroke Riskometer demonstrated superior psychometric properties in assessing stroke risks in several studies compared to other available methods and applications^6,9–11^. Since its introduction in 2014, this application has become increasingly popular and widely used^11^. However, before this current study, its cross-cultural validation was not conducted using robust psychometric methodology. Therefore, this study was novel in using G-Theory to examine psychometric properties of the Stroke Riskometer across different countries. The findings of this study have added evidence for reliability of the Stroke Riskometer across countries in estimating stroke risks.

A D-study was conducted in an attempt to evaluate reliability of the Stroke Riskometer for each country, as well as the impact of each country on the overall reliability of the Stroke Riskometer. Similar to another cross-cultural validation study that applied G-Theory to evaluate the positive and negative syndromes scale for assessment of psychotic symptoms across different countries^23^, we have also iteratively removed each country from the analyses and observed the overall reliability estimates. If by removing a specific country the overall reliability would increase, this would provide indirect evidence that there may be a negative impact on the reliability of assessment in this specific country. However, there were no noticeable improvements of the overall reliability by removing countries in our study. Together, these findings demonstrated that the Stroke Riskometer is reliable to use for estimating the risk of stroke in different countries in the world.

Moreover, other D-study analyses removing one or more item(s) at a time were conducted in an effort to examine the impact of each individual item and group of items on the overall reliability of the Stroke Riskometer. This methodology is the best practice used by other studies employing G-Theory approach and demonstrated their usefulness in optimizing measurements^17^. Our results indicated that removing individual items did not improve reliability of the Stroke Riskometer and made it worse in comparison to reliability estimates of the full scale. This finding showed that the Stroke Riskometer is the most reliable in measuring stroke risks using its current assessment format and included risk factors.

CTT is currently the dominant statistical method for examining reliability of an assessment. Earlier studies have employed CTT to estimate the reliability of the Stroke Riskometer^6,9^. However, it seems that there has not been any research applying the CTT method to evaluate the cross-cultural validation for this application. There also seems to be no research using the CTT approach for examining this validation for different countries in a single analysis. Literature reviews indicated that most CTT studies that conducted cross-cultural validation of psychometric instruments were based on internal consistency (Cronbach’s alpha) and/or test–retest reliability coefficients from a specific country^25,26^. Therefore, CTT methods are unable to evaluate error sources due to the effect of individual facets such as items, countries, and their interactions together in a single analysis. The application of G-Theory in this study demonstrated its superiority to CTT in assessing the reliability of the Stroke Riskometer. The G-Theory method used in this study estimated precisely all possible influences on reliability (country error, item nested in country error, and error in interactions between item, country and/or person) simultaneously, providing a more rigorous evaluation of the overall reliability, permitting generalizability of the Stroke Riskometer scores across sample population and countries.

Normally, in a generalizability study, the item facet is fixed because the same items of the assessment are used across all participants and all circumstances^16,17^. However, in this study the item facet was set at infinite because the items used in different countries were translated into different languages. In the literature facets are commonly defined as infinite if a researcher is interested in generalizing their findings over a facet, for example, the country facet in the current study^23,24^. Essentially, generalizing assessment scores over countries is the primary purpose of cross-cultural validation studies. Therefore, the current observational design was applied for the purpose of examining how translation of Stroke Riskometer items into different languages would impact on reliability. Our results show that the Stroke Riskometer’s scores were also generalizable across the set of translated assessment items and countries, which suggests that appropriately translating these items into different languages of other countries not included in the current study may be equally reliable.

### Strengths and limitations

The main strength of the study was the relatively large and ethnically diverse/mixed sample of the study population, including Caucasians (mostly from Australia, France, Italy, New Zealand, Russia, Ukraine, United Kingdom, the United States) and non-Caucasian people (Brazil, India, Kazakhstan, Malaysia, Nigeria). However, with this large sample size we were not able to evaluate the generalizability of the Stroke Riskometer stratified by ethnicity because the ethnicity-specific data were unbalanced between countries with many sample sizes being too small to ensure robustness of G-analyses. Future studies should replicate these analyses with adequate samples of culturally diverse participants including a larger number of countries. Generally, our results are in line with the notion that the effect of generalizability analysis depends on the number of countries, assessment items and the study population. Therefore, excluding one country or item from a very large population would have negligible impact on the analysis while excluding a country or item from a much smaller population might have a significant impact. We acknowledge that cultural beliefs might also influence the interpretation of and/or responses to the translated questions and such influence could be investigated by measuring differences in cultural beliefs. However, this study did not include such measures, which is a limitation that can be addressed in future research. To the best of our knowledge there is no research literature suggesting additional cross-national variation of the app that needs to be tested at this point. Should such evidence arise, the authors are committed to test it in a future study.

## Conclusion

In conclusion, the findings of this study indicate that the Stroke Riskometer reliably captures stroke risk across countries. Thus, users can rely on its ability to assess their stroke risks, but as it is a novel stroke prevention strategy, the Stroke Riskometer should continue to evolve and more research is needed to further enhance precision and validity of this useful instrument.

## Methods

### Participants

**Figure 4:**
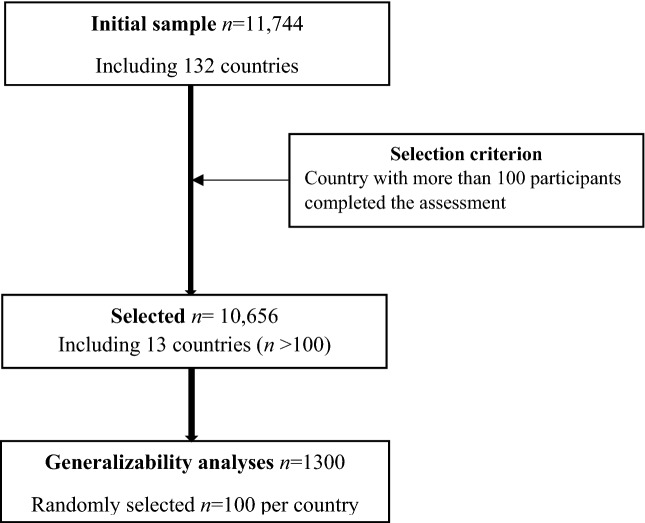
Consort flow diagram of the study sample selection for Generalizability analyses of the Stroke Riskometer.

Participants (n = 11,744) from 132 countries worldwide completed the Stroke Riskometer questionnaire. In order to have an adequate sample size for the generalizability analyses (G-analyses), we firstly selected countries where there were more than 100 completed stroke risk assessments. There were thirteen countries meeting this selection criterion (namely Australia, Brazil, France, India, Italy, Kazakhstan, Malaysia, New Zealand, Nigeria, Russia, Ukraine, United Kingdom, and the United States). We then randomly selected an equal number of 100 participants for each selected country. The study sample finally consisted of 1300 participants with 100 in each country, as shown in Fig. 4. Participants’ ages ranged from 20 to 89 (mean = 46.28; standard deviation SD = 14.96). There were 680 (52.3%) male and 620 (47.7%) female participants. Ethnic groups included 64.2% European, 11.2% Asian, 8.1% Indian, 1.0% Polynesian, and 15·5% others. The study was approved by the Auckland University of Technology Ethics Committee (AUTEC Ref.#19/236) and carried out in accordance with the AUTEC guidelines and regulations. All participants provided their informed consent to participate in this research. We have no evidence to believe that people who provided the data were vulnerable since informed consent was obtained from them as they entered the data remotely over the internet. None of these participants contacted us after they provided the data.

### Measure

The Stroke Riskometer^6^ consists of 21 assessment items including three demographic questions (i.e. age, sex, and ethnicities), two physical questions (height and weight), and 16 other questions identifying major risk factors of stroke including blood pressure. Dependent upon each individual item, some items use two categorical response options (0–1) and some three (0–2). For some items, a cut-off point is used to determine ordinal category. For instance, item 12 is recoded into 0 for participants who had blood pressure less than 120, and 1 for those who had that of 120 or above. G-analyses were conducted on the core 16 questions in the Stroke Riskometer included in Table 4. All 16 questions about physiological risk factors, including blood pressure, were also considered as risk factors, while age, sex, race/ethnicity, height and weight did not contribute to measurement error because significant variability of these factors is not expected at the individual level. Therefore, stratification by sex and age were omitted because these variables cannot vary within an individual and cannot be interpreted differently in different countries. However, other physiological risk factors, including blood pressure may vary substantially within each individual and may substantially increase error variance.

**Table 4:** Sixteen stroke riskometer questions used in G-analyses.

Question	Content
1	Do you currently smoke, or have you smoked over the past year?
2	Do you drink more than 1 standard alcoholic drink a day?
3	Do you eat at least 6 servings of fruits and/or vegetables a day?
4	Have you experienced significant mental/emotional stress or depression in the past year?
5	Did your mother or father have a stroke or heart attack before the age of 65?
6	What is your systolic blood pressure (the higher of the 2 numbers of your blood pressure reading)?
7	Are you using blood pressure lowering medication?
8	Have you ever been told by a doctor that you have diabetes?
9	Have you ever been told by a doctor you have any kind of heart disease?
10	Have you ever been told by a doctor that you have an enlarged heart?
11	Have you ever been told by your doctor that you have irregular heartbeats (atrial fibrillation)?
12	Have you ever been told by a doctor that you have a cognitive problem or dementia?
13	Do you or anyone close to you think you have poor memory?
14	Have you ever been told by a doctor that you have a traumatic brain injury?
15	Have you ever been told by a doctor that you’ve had a stroke or transient ischaemic attack (mini stroke)?
16	What is your systolic blood pressure (the higher of the 2 numbers of your blood pressure reading)?

### Data analyses

EduG 6.1-e software (Swiss Society for Research in Education Working Group 2006) was used to conduct Generalizability analyses. Both G-study and D-study used the two-facet nested design where facet item (I) is nested in facet country (C) and person (P) was the object of measurement (differentiation facet), expressed as P × (I:C)^15,27,28^. In this study design, the I facet was infinite as were the P and C facets because the set of items used in different countries were translated into different languages. The facet P reflected true differentiation amongst persons and was not a source of error in a generalizability study meaning that after controlling for person variance (P), all other sources of error variances were accounted for 100%^29^.

Generally, for the two-facet design, expressed as P × I × C, the effects for all facets were presented by *X* which was the observed score of a person on a particular item across countries and were obtained as below^15,24^:

$$X= \mu +{X}_{p}+{X}_{i}+{X}_{c}+{X}_{pi}+{X}_{pc}+{X}_{ic}+{X}_{pic},$$

$\text{where}: \mu$ = grand mean of *X, X*_p_ = μ_p_ − μ (person effect), *X*_i_ = μ_i_ – μ (item effect), *X*_c_ = μ_c_ – μ (country effect), *X*_pi_ = μ_pi_ – μ_p_ – μ_i_ + μ (person × item effect), *X*_pc_ = μ_pc_ − μ_p_ − μc + μ (person × country effect), *X*_ic_ = μ_ic_ − μ_i_ − μ_c_ + μ (item × country effect), *X*_pic_ = μ_pic_ − μ_pi_ − μ_pc_ − μ_ic_ + μ_p_ + μ_i_ + μ_c_ − μ (residual/person × item × country effect).

Each of the effects has estimated variance components, which were possible sources of error that might impact measurement and were calculated as follow with MS stands for the mean of effect squared and n represents a facet sample size^15,24^:

- Person variance component: *σ*^*2*^_*p*_ = (MS_p_ − MS_pi_ − MS_pc_ + MS_pic_)/n_i_n_c_
- Item variance component: *σ*^*2*^_*i*_ = (MS_i_ − MS_pi_ − MS_ic_ + MS_pic_)/n_p_n_c_
- Country variance component: *σ*^*2*^_*c*_ = (MS_c_ − MS_ic_ − MS_pc_ + MS_pic_)/n_i_n_c_
- Person × Item variance component: *σ*^*2*^_*pi*_ = (MS_pi_ − MS_pic_)/n_c_
- Person × Country variance component: *σ*^*2*^_*pc*_ = (MS_pc_ − MS_pic_)/n_i_
- Item × Country variance component: *σ*^*2*^_*ic*_ = (MS_ic_ − MS_pic_)/n_p_
- Person × Item × Country variance component: *σ*^*2*^_*pic*_=MS_pic_.

However, this study was designed as the two-facet nested design with item facet nested in country facet and in this case the relevant variance components were obtained as follows^28^:

- Item nested in Country variance component: *σ*^*2*^_*(i:c)*_ = *σ*^*2*^_*i*_ + *σ*^*2*^_*pc*_
- Residual/Person × Item nested in Country variance component: *σ*^*2*^_*p*x*(i:c)*_ = *σ*^*2*^_*ic*_ + *σ*^*2*^_*pic*_

There are two reliability coefficients used in G-Theory: relative G-coefficient (*Gr*) and absolute G-coefficient (*G*_*a*_). The relative model of measurement is a norm-referenced approach, which is based on a relative error variance (${\sigma }_{\delta }^{2}$). According to this model, a person’s assessment score is compared against the scores of others^30^ and thus *G*_*r*_ is computed as below^15,28^:

$${G}_{r}=\frac{{\upsigma }_{\mathrm{p}}^{2}}{{\upsigma }_{\mathrm{p}}^{2}+ {\upsigma }_{\updelta }^{2}};\mathrm{where} \; {\sigma }_{\delta }^{2}={\sigma }_{pc}^{2}+{\sigma }_{p\mathrm{x}(i:c)}^{2}.$$

The absolute model of measurement is based on a criterion-referenced approach, where a person’s score is compared against some agreed-upon absolute standard^30^. *Ga* accounts for an absolute error variance (*σ*^*2*^_*Δ*_) which involves interaction between country (C) and item nested in country (I:C) that possibly influences an absolute measurement indirectly^24,27,29^:

$${G}_{a}=\frac{{\upsigma }_{\mathrm{p}}^{2}}{{\upsigma }_{\mathrm{p}}^{2}+ {\upsigma }_{\Delta }^{2}};\mathrm{where }\; {\sigma }_{\Delta }^{2}={\sigma }_{c}^{2}+{\sigma }_{pc}^{2}+{\sigma }_{(i:c)}^{2}+{\sigma }_{p\mathrm{x}(i:c)}^{2}.$$

Both _*G*_ coefficients (i.e., *G*_*r*_ and *G*_*a*_) reflect reliability of a measurement with the measurement design where the differentiation facet is person (P). Specifically*, Ga* uses more stringent criteria and coefficients above 0.70 reflect acceptable reliability^17^, while *Gr* of 0.80 or higher indicates good reliability of assessment scores^29^.

In the D-study, variance components were computed for each country and for further analyses to evaluate the impact of each country on the reliability of the Stroke Riskometer. In order to optimize reliability of the measurement we also examined if modification of measurement design (e.g., removing an item) may lead to enhanced reliability.
