# Author Correction: Cross-cultural validation of the stroke riskometer using generalizability theory

**DOI:** 10.1038/s41598-021-00028-9

**Published:** 2021-10-11

**Authors:** Oleg Medvedev, Quoc Cuong Truong, Alexander Merkin, Robert Borotkanics, Rita Krishnamurthi, Valery Feigin

**Affiliations:** 1 School of Psychology, Faculty of Arts and Social Sciences, University of Waikato, Private Bag 3105, Hamilton, 3240 New Zealand; 2 School of Clinical Sciences, Auckland University of Technology, Auckland, New Zealand

Correction to: *Scientific Reports* 10.1038/s41598-021-98591-8, published online 24 September 2021

The original version of this Article contained an error in the spelling of the author Quoc Cuong Truong which was incorrectly given as Quoc Truong. The original Article has been corrected.

